# Long noncoding RNA HOXA-AS2 represses P21 and KLF2 expression transcription by binding with EZH2, LSD1 in colorectal cancer

**DOI:** 10.1038/oncsis.2016.84

**Published:** 2017-01-23

**Authors:** J Ding, M Xie, Y Lian, Y Zhu, P Peng, J Wang, L Wang, K Wang

**Affiliations:** 1 Department of Oncology, Second Affiliated Hospital, Nanjing Medical University, Jiangsu, PR China; 2 Center for Reproduction and Genetics, Suzhou Municipal Hospital, Nanjing Medical University Affiliated Suzhou Hospital, Jiangsu, PR China

## Abstract

Long noncoding RNAs (lncRNAs) have received increased attention as a new class of functional regulators involved in human carcinogenesis. HOXA cluster antisense RNA 2 (HOXA-AS2) is a 1048-bp lncRNA located between the *HOXA3* and *HOXA4* genes in the HOXA cluster that regulates gene expression at a transcription level. HOXA-AS2 is previously found to be overexpressed in gastric cancer (GC) and promotes GC cells proliferation. However, its potential role and molecular mechanism in colorectal cancer (CRC) are not known. Here, we identified that HOXA-AS2 is significantly upregulated in CRC tissue. In addition, increased HOXA-AS2 expression is associated with a larger tumor size and an advanced pathological stage in CRC patients. HOXA-AS2 knockdown significantly suppressed proliferation by blocking the G1/S transition and caused apoptosis of CRC cells *in vitro* and *in vivo*. The mechanistic investigations showed that HOXA-AS2 could interact with EZH2 (enhancer of zeste homolog 2), LSD1 (lysine specific demethylase 1) and recruit them to p21 (CDKN1A), KLF2 promoter regions to repress their transcription. Furthermore, the rescue experiments demonstrated that HOXA-AS2 oncogenic function is partly through regulating p21. In conclusion, our data suggest that HOXA-AS2 may function as an oncogene by modulating the multiple genes expression involved in CRC proliferation, and also provides a potential target for CRC therapy.

## Introduction

Colorectal cancer (CRC) is one of the top three culprits of common malignancies worldwide, with over 1.2 million new cases diagnosed each year.^1, 2, 3^ Although considerable headway in CRC diagnosis and therapy has been achieved in the past decade, CRC morbidity and mortality remain high, especially in the developed world.^4^ As is known to all, CRC is a complicated disease involving multiple genomic variation and biological processes.^5^ Therefore, further study of the molecular mechanisms must be conducted to gain a deeper understanding of CRC.

The Human Genome Project revealed that only small portions of the genome encode proteins, whereas the vast majority (~97%) of the human genome is transcribed into noncoding RNAs.^6, 7, 8^ Long noncoding RNAs (lncRNAs) are a class of newly discovered noncoding RNA molecules and have attracted the attention of many researchers given their abnormal expression in human diseases.^9, 10^ Considerable evidence has suggested that lncRNAs are key oncogenes^11, 12^ or tumor suppressors^13^ in human carcinogenesis and are increasingly recognized as future diagnostic, therapeutic or prognostic cancer biomarkers, including for CRC.^14, 15, 16^ Our previous studies also demonstrated that downregulation of lncRNA BANCR (BRAF activated noncoding RNA) may contribute to the proliferation of CRC cells, at least in part, through the regulation of p21 protein;^17^ lncRNA LOC554202 could induce apoptosis via the caspase cleavage cascades in CRC cells.^18^

The underlying molecular mechanisms involved in lncRNA interactions are complex and diverse. These RNAs may function as signals, decoys, guides or scaffolds^19, 20^ that participate in intertwined gene regulatory networks at various levels.^21^ Typical lncRNAs can coordinate histone modifications by binding to various histone modification enzymes. For example, the 5′ domain of lncRNA HOTAIR binds to PRC2 (Polycomb Repressive Complex 2), whereas the 3′ domain binds to the LSD1 (lysine specific demethylase 1)/CoREST/REST complex, which acts as a modular scaffold.^22^ PRC2, which consists of EZH2 (enhancer of zeste homolog 2), SUZ12 (suppressor of zeste 12) and EED (embryonic ectoderm development), is a methyltransferase for histone H3 lysine 27 trimethylation (H3K27me3).^23^ LSD1 is a demethylase that mediates the enzymatic demethylation of histone H3 lysine 27 dimethylation (H3K4me2).^24^

HOXA cluster antisense RNA 2 (HOXA-AS2) is a 1048-bp lncRNA that serves as an apoptosis repressor in all-trans retinoic acid-treated NB4 promyelocytic leukemia cells.^25^ In previous studies, we also demonstrated that HOXA-AS2 promotes gastric cancer cells proliferation by epigenetically silencing p21, PLK3 and DDIT3 expression.^26^ However, little is known concerning its potential role in CRC development and progression. In this study, we sought to assess the function and molecular mechanisms of HOXA-AS2 in CRC. We found that HOXA-AS2 expression is upregulated in CRC tissues and cell lines and is tightly associated with increased tumor size and an advanced pathological stage in CRC patients. Ectopic expression of HOXA-AS2 in CRC cells significantly promoted cell proliferation and inhibited apoptosis *in vivo* and *in vitro*. We further demonstrated that HOXA-AS2 functions as a modular scaffold of histone modification complexes via binding with EZH2 and LSD1 to silence p21 and KLF2 expression. These results suggest that HOXA-AS2 may have a significant role in the progression of CRC and could serve as a new therapeutic target.

## Results

### HOXA-AS2 expression is increased in human CRC tissues and cell lines

**Figure 1:**
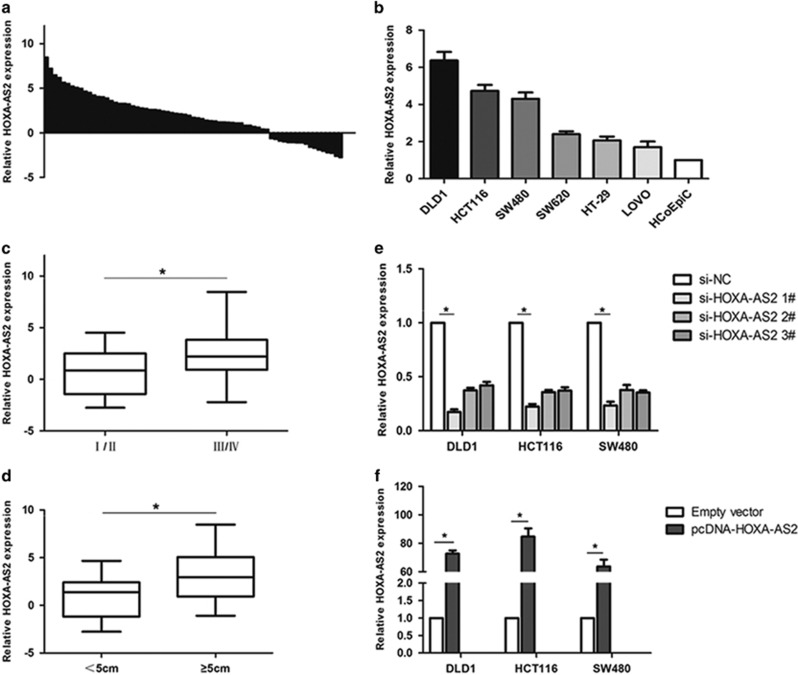
Relative expression of HOXA-AS2 in colorectal cancer tissues and cells compared with adjacent normal tissues and normal colonic epithelial cells. (**a**) The relative expression of HOXA-AS2 in colorectal cancer tissues (*n*=69) compared with corresponding non-tumor tissues (*n*=69). HOXA-AS2 expression was examined by qRT–PCR and normalized to GAPDH expression. The results are presented as the fold-change in tumor tissues relative to normal tissues (presented as −ΔΔCT). (**b**) HOXA-AS2 expression was assessed by qRT–PCR in colorectal cancer cell lines (DLD1, HCT116, SW480, SW620, HT-29 and LOVO) and compared with the normal human colonic epithelial cell line (HCoEpiC). (**c**, **d**) The data are presented as the relative expression levels in tumor tissues. HOXA-AS2 expression was significantly increased in patients with a higher pathological stage and larger tumors. (**e**, **f**) qRT–PCR analysis of HOXA-AS2 expression levels following the treatment of DLD1, HCT116 and SW480 cells with si-HOXA-AS2 or pCDNA-HOXA-AS2 or the negative control. *P<0.05.

We first examined HOXA-AS2 expression levels in 69 CRC tissue samples and matched adjacent normal tissues by performing quantitative real-time PCR (qRT–PCR) analysis. In 48 cases, HOXA-AS2 expression was at least twofold increased (*P*<0.05) in the CRC tissues compared with paired normal tissues (Figure 1a). Increased HOXA-AS2 expression was also validated in CRC cells, including DLD1, HCT116, SW480, SW620, HT-29 and LOVO cells (*P*<0.05, Figure 1b). Furthermore, to understand the significance of HOXA-AS2 expression in CRC, the clinicopathological features are summarized in Table 1. As shown in Figures 1c and d, upregulated HOXA-AS2 expression is closely related to increased tumor size, advanced TNM stage and lymph node metastasis in the CRC patients, implying that HOXA-AS2 is tightly associated with tumorigenesis and CRC progression.

### SiRNA selection for HOXA-AS2 silencing or overexpression in CRC cells

To observe the functional relevance of HOXA-AS2 in CRC cells, we chose the DLD1, HCT116 and SW480 cell lines for further studies because they exhibited the highest expression levels (Figure 1b). The qRT–PCR analysis was used to detect the transfection efficiency (Figures 1e and f). Of the three siRNAs, si-HOXA-AS2 1# and 2# demonstrated the better silencing capacity. HOXA-AS2 expression was increased in all the three CRC cell lines compared with the negative control following transfection with pCDNA-HOXA-AS2. Thus, we selected si-HOXA-AS2 1# and pCDNA-HOXA-AS2 in all further experiments for HOXA-AS2 silencing or overexpressing, respectively. To avoid the off-target effects of siRNAs, we also use si-HOXA-AS2 2# in the experiments for exploring the biological function of HOXA-AS2 (Supplementary Figure S1).

### HOXA-AS2 promotes CRC cell proliferation *in vitro*

**Figure 2:**
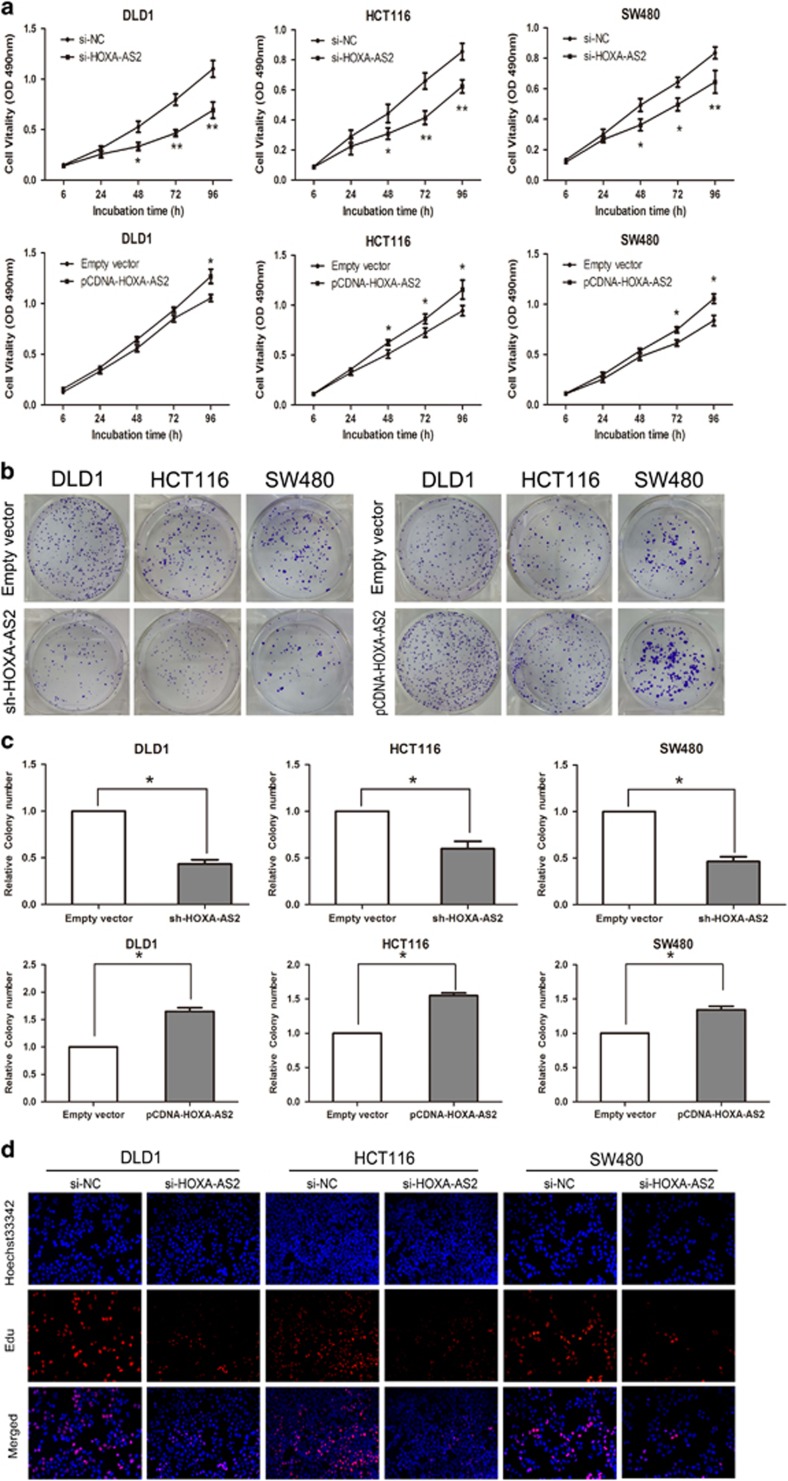
HOXA-AS2 promotes CRC cells proliferation *in vitro.* (**a**) An MTT assay was performed to determine the proliferation of DLD1, HCT116 and SW480 cells following treatment with sh-HOXA-AS2, pCDNA-HOXA-AS2 or empty vector. The data represent the means±s.d. from three independent experiments. (**b**,**c**) Colony-forming growth assays were performed to determine the proliferation of CRC cells. The colonies were counted and captured. (**d**) Proliferating CRC cells were labeled with Edu. The Click-it reaction revealed Edu staining (red). Cell nuclei were stained with Hoechst 33342 (blue). The images are representative of the results obtained. *P<0.05 and **P<0.01.

The MTT assay was performed to assess the potential biological function of HOXA-AS2 in CRC cell proliferation. The vitality of DLD1, HCT116 and SW480 cells transfected with siRNA-HOXA-AS2 was significantly inhibited compared with control groups, whereas HOXA-AS2 overexpression enhanced the cell growth capacity (*P*<0.05, Figure 2a). To determine whether HOXA-AS2 expression alteration also affects the tumorigenicity of CRC cells, colony-formation assays were performed. These data suggest that the CRC cell colony numbers significantly decreased following knockdown of HOXA-AS2 but increased in CRC cells overexpressing HOXA-AS2 (*P*<0.05, Figures 2b and c). Furthermore, the Edu (ethynyl deoxyuridine; red)/Hoechst (blue) immunostaining also confirmed this result (Figure 2d). Taken together, these findings indicate that HOXA-AS2 may function as an oncogene and markedly increases CRC function and tumorigenicity.

### Downregulation of HOXA-AS2 promotes G1 arrest and causes apoptosis in CRC cells *in vitro*

**Figure 3:**
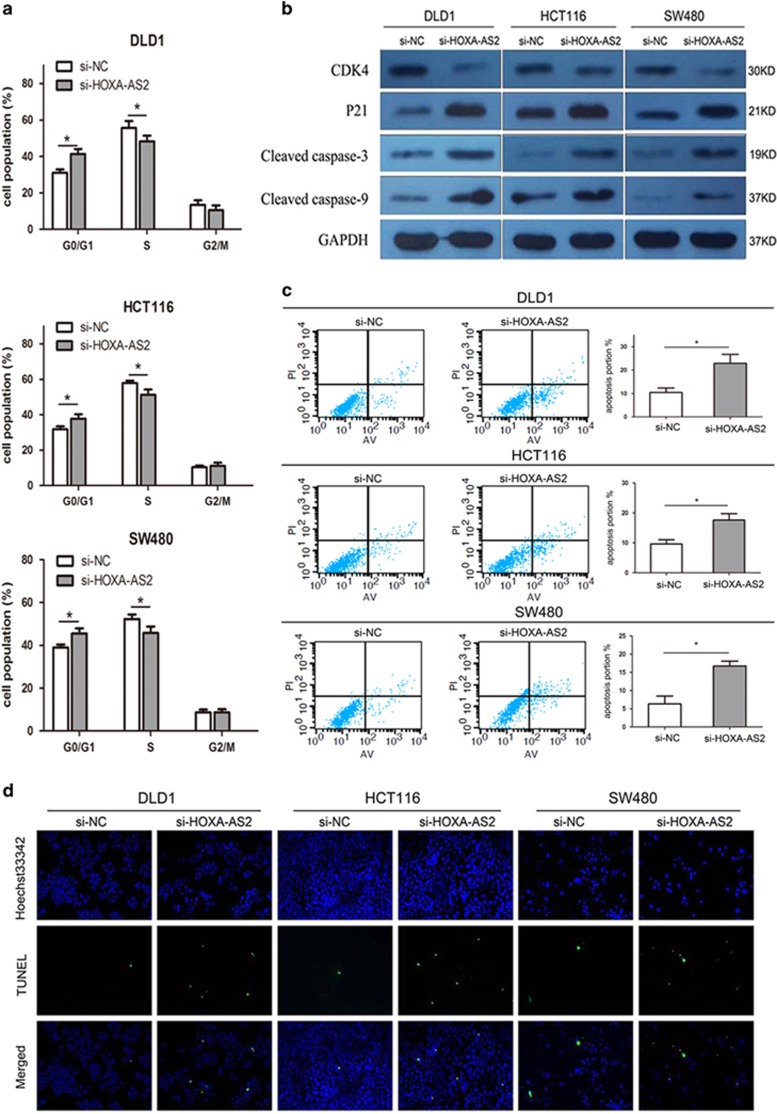
Downregulation of HOXA-AS2 promotes G1 arrest and causes apoptosis in CRC cells *in vitro.* (**a**) The bar chart represents the percentage of cells in G0/G1, S or G2/M phase, as indicated. (**b**) Western blot analysis of CDK4, p21 and cleaved caspase-3 and cleaved caspase-9 after si-HOXA-AS2 or si-NC transfection in DLD1, HCT116 and SW480 cells. GAPDH protein was used as an internal control. (**c**) The percentage of apoptotic cells was determined by flow cytometric analysis. The data represent the mean±s.d. from three independent experiments. (**d**) The level of apoptosis in CRC cells after transfection with si-HOXA-AS2 or si-NC as determined by TUNEL staining. *P<0.05. NC, negative control.

To assess whether the pro-proliferative effects of HOXA-AS2 on the CRC cells are mediated by promoting cell cycle progression, we examined cell cycling in CRC cells by flow cytometry. As shown in Figure 3a, silencing HOXA-AS2 promoted a significant arrest in the G0/G1-phase, with an obvious reduction in the number of cells in the S-phase (*P*<0.05). Consistent with this finding, the results of western blot analysis showed that the protein levels of CDK4 (cyclin-dependent kinase 4) and p21 were significantly changed in si-HOXA-AS2-treated cells (Figure 3b), confirming that HOXA-AS2 is involved in cell cycle regulation.

Moreover, flow cytometric analysis was utilized to investigate whether apoptosis regulation is a potential contributing factor to cell growth progress induced by HOXA-AS2. The results demonstrated that the silencing of HOXA-AS2 expression significantly increased in CRC cell apoptosis (*P*<0.05, Figure 3c). Similarly, microscopic analysis of TUNEL (terminal deoxynucleotidyl transferase-mediated dUTP nick end labeling) staining in CRC cells also revealed that HOXA-AS2 knockdown resulted in an increased number of apoptotic cells compared with controls (*P*<0.05, Figure 3d). In addition, western blot analysis demonstrated that cleaved caspase-3 and cleaved caspase-9 proteins were increased in si-HOXA-AS2-treated CRC cells (*P*<0.05, Figure 3b). These investigations suggest that HOXA-AS2 exerts critical influences on CRC cells by affecting both the cell cycle and apoptosis.

### HOXA-AS2 epigenetically silences p21 and KLF2 transcription by binding to EZH2 and LSD1

**Figure 4:**
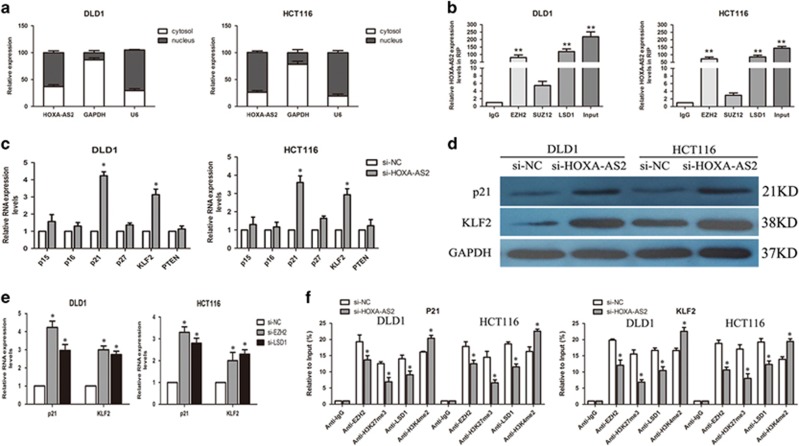
HOXA-AS2 epigenetically silences p21 and KLF2 transcription by binding to EZH2 and LSD1. (**a**) qRT–PCR analysis of HOXA-AS2 nuclear and cytoplasmic expression levels in DLD1 and HCT116 cells. U6 was used as a nucleus marker, and GAPDH was used as a cytosol marker. (**b**) RIP experiments were performed in DLD1 and HCT116 cells, and the coprecipitated RNA was subjected to qRT–PCR for HOXA-AS2. The fold enrichment of HOXA-AS2 in EZH2/SUZ12/LSD1 RIP is relative to its matched IgG control. (**c**) The levels of p15, p16, p21, p27, KLF2 and PTEN mRNA were determined by qRT–PCR when knockdown of HOXA-AS2. (**d**) The p21 and KLF2 protein levels were determined by western blot in HOXA-AS2 knockdown in DLD1 and HCT116 cells. (**e**) The p21 and KLF2 expression levels were determined by qRT–PCR when knockdown of EZH2 or LSD1 in DLD1 and HCT116 cells. (**f**) ChIP-qRT–PCR of EZH2/LSD1 occupancy and H3K27me3/H3K4me2 binding in the p21 and KLF2 promoters in DLD1 and HCT116 cells treated with si-HOXA-AS2 (48 h) or si-NC; IgG as a negative control. Error bars indicate mean±s.e.m. *P<0.05, **P<0.01. ChIP, chromatin immunoprecipitation; NC, negative control.

Multiple molecular mechanisms have been proposed to explain lncRNA-mediated gene expression. HOXA-AS2 is previously found to be overexpressed in gastric cancer and promotes its cell proliferation;^26^ however, its molecular mechanism and downstream targets involving in regulation of CRC cells phenotype is not known. To investigate the potential mechanism and downstream targets of HOXA-AS2 in CRC cells, we first analyzed its distribution in the CRC cells and found that HOXA-AS2 mostly located in the nucleus (*P*<0.05, Figure 4a), implying that HOXA-AS2 may be involved in transcriptional regulation.

To further investigate whether HOXA-AS2 could bind to histone modification enzymes, we performed the RNA immunoprecipitation (RIP) assay. The results showed that HOXA-AS2 could directly binds with EZH2 and LSD1 in DLD1 and HCT116 cells and does not bind with SUZ12 (Figure 4b). Typical lncRNA HOTAIR was used as a positive control (Supplementary Figure S2). The RNA-pulldown experiments also confirmed this result (Supplementary Figure S3). Then we selected some important EZH2 or LSD1 underlying targets and hypothesized that they may also involve in the contributions of HOXA-AS2 to CRC development. The qRT–PCR results showed that inhibition of HOXA-AS2 expression led to increased p21 and KLF2 expression, whereas in the other genes, there was no siginificant difference (*P*<0.05, Figure 4c). Consistent with this finding, the results of western blot analysis showed that the protein levels of p21 and KLF2 were significantly changed in si-HOXA-AS2-treated cells (*P*<0.05, Figure 4d). Meanwhile, the expression of p21 and KLF2 were increased after transfection of si-EZH2 or si-LSD1, which indicated that p21 and KLF2 could be HOXA-AS2 downstrean targets in CRC cells (*P*<0.05, Figure 4e). Furthermore, chromatin immunoprecipitation analysis demonstrated that EZH2 directly binds to p21 and KLF2 promoter regions and induces H3K27me3 modification, whereas LSD1 directly couples with their promoter regions and enhances H3K4me2 modification in the CRC cells (*P*<0.05, Figure 4f). These data suggested that HOXA-AS2 promotes CRC cell proliferation in a manner that is dependent on regulation of p21 and KLF2 expression not only through binding to EZH2 but also by combining with LSD1.

### P21 silencing potentially involves the oncogenic function of HOXA-AS2

**Figure 5:**
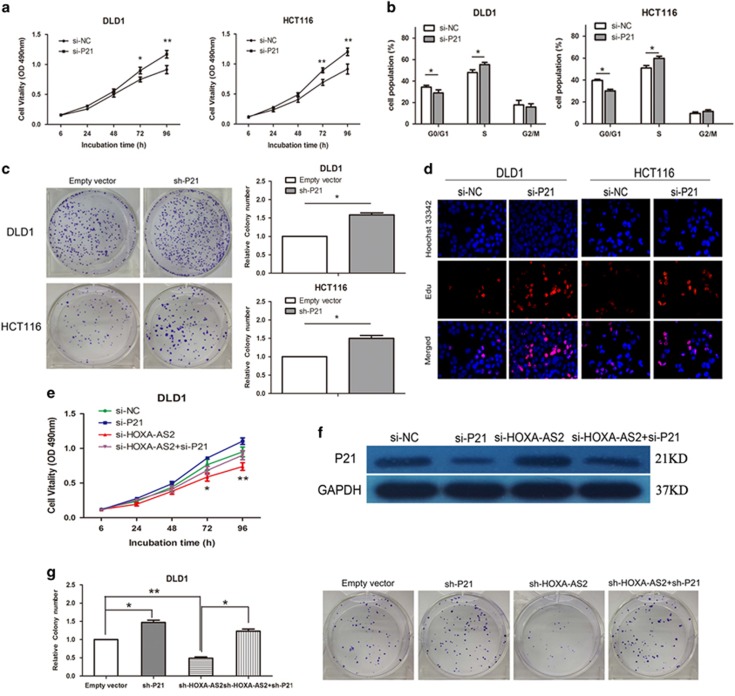
Silencing p21 potentially involves the oncogenic function of HOXA-AS2. (**a**) An MTT assay was performed to determine DLD1 and HCT116 cell proliferation following treatment with si-p21 or si-NC. The data represent the means±s.d. from three independent experiments. (**b**) The bar chart represents the percentage of cells in G0/G1, S or G2/M phase, as indicated. (**c**) Colony-forming growth assays were performed to determine CRC cell proliferation. The colonies were counted and captured. (**d**) Proliferating CRC cells were labeled with Edu. The Click-it reaction revealed Edu staining (red). Cell nuclei were stained with Hoechst 33342 (blue). The images are representative of the results obtained. (**e**, **g**) MTT and colony-formation assays were used to determine the cell viability for si-HOXA-AS2 and si-p21 co-transfected DLD1 cells. (**f**) Western blot analysis of p21 after si-HOXA-AS2 and si-p21 co-transfection in DLD1 cells. GAPDH protein was used as an internal control. Experiments were performed in triplicate. Error bars indicate mean±s.e.m. *P<0.05 and **P<0.01. NC, negative control.

To determine whether p21 is involved in the HOXA-AS2-induced increase in CRC cell proliferation, MTT and colony-formation assays were performed in DLD1 and HCT116 cells (Figures 5a). The results indicated that silenced p21 expression significantly promoted cell proliferation. In addition, the Edu assay also confirmed this result (Figure 5d). Moreover, flow cytometry analysis indicated that reduced p21 expression decreases the G0/G1-phase arrest (*P*<0.05, Figure 5b). Furthermore, to validate whether HOXA-AS2 regulates CRC cell proliferation by silencing p21 expression, rescue assays were performed. DLD1 cells were co-transfected with si-HOXA-AS2 and si-p21, and the MTT and colony-formation assay results indicated that co-transfection partially rescues si-HOXA-AS2-damaged proliferation ability (*P*<0.05, Figures 5e). Moreover, the western blot results indicate that co-transfection reduces the upregulated expression of p21 induced by the knockdown of HOXA-AS2 (*P*<0.05, Figure 5f). These results indicated that the effect of HOXA-AS2 on CRC partially involves targeting p21.

### Knockdown of HOXA-AS2 inhibits CRC tumorigenesis *in vivo*

**Figure 6:**
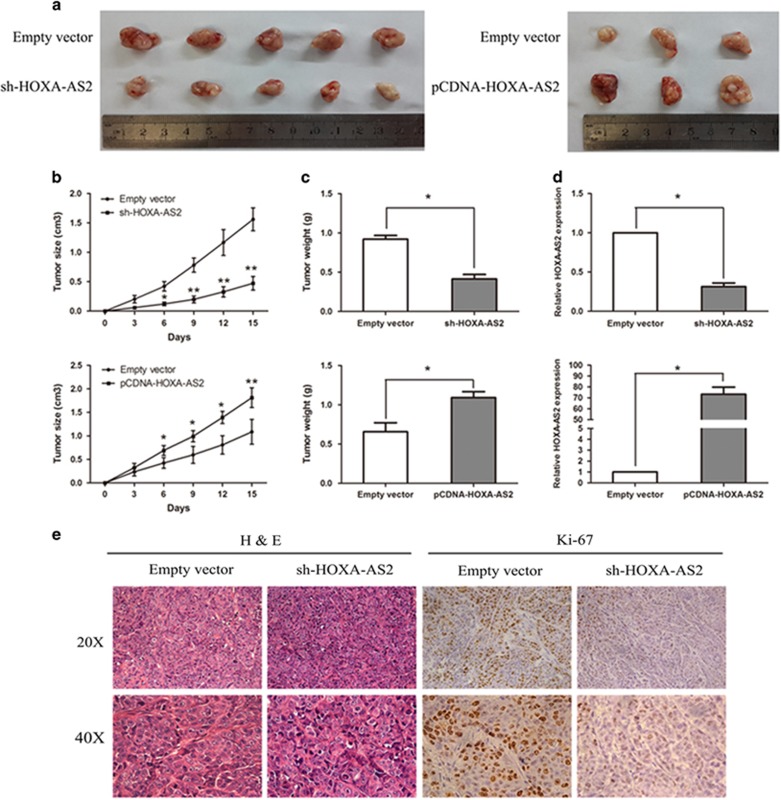
Knockdown of HOXA-AS2 inhibits CRC tumorigenesis *in vivo.* (**a**) The total number of tumors after removal from the mice. (**b**) The tumor volumes were calculated every 3 days after inoculation. (**c**) The tumor weights after the tumors were harvested. The data represent the means±s.d. (**d**) qRT–PCR analyses indicated that the HOXA-AS2 expression was significantly increased *in vivo*. (**e**) Representative images of hematoxylin and eosin (H&E) and immunohistochemical staining of the tumor. Immunohistochemical (IHC) revealed a downregulation of the proliferation index Ki-67. *P<0.05 and **P<0.01.

To determine the possibility that HOXA-AS2 expression could affect CRC tumorigenesis *in vivo*, sh-HOXA-AS2 or pCDNA-HOXA-AS2, as well as negative control-transfected HCT116 cells were subcutaneously inoculated into male nude mice. Fifteen days after injection, the tumors formed in the sh-HOXA-AS2 group were dramatically smaller than those in the negative control group, whereas the tumors of the pCDNA-HOXA-AS2-transfected group were obviously larger compared with the empty vector group (*P*<0.05, Figure 6a). Similarly, the tumor growth rate and the average tumor weight in the sh-HOXA-AS2 group was significantly reduced compared with the control group, and the pCDNA-HOXA-AS2 group exhibited the opposite results (*P*<0.05, Figures 6b and c). Moreover, HOXA-AS2 expression remained markedly reduced in the sh-HOXA-AS2 group, as well as increased in the pCDNA-HOXA-AS2 group (*P*<0.05, Figure 6d). The immunohistochemical assay results show that the tumors developed from sh-HOXA-AS2 cells displayed karyopyknosis, alterations in shape and reduced Ki-67 staining compared with tumors formed from empty vector-transfected cells (Figure 6e). These results indicate that HOXA-AS2 is significantly associated with CRC cell proliferation *in vivo*.

## Discussion

With advances in tiling array and sequencing technologies, it has been revealed that the majority (~97%) of human genome sequences is transcribed into noncoding RNAs (ncRNAs). These ncRNAs can be divided into two groups according to length, including short ncRNAs (<200nt) and long ncRNAs (>200 nt).^6, 7^ A growing body of evidence has demonstrated that lncRNAs have an important role in the pathogenesis of cancer.^27, 28, 29^ Nonetheless, it is obvious that the precise molecular mechanisms underlying the CRC remain largely unclear.

We herein uncover a novel carcinogenic role of HOXA-AS2 in the CRC cells. We first found that HOXA-AS2 was significantly upregulated in the CRC tissues compared with the adjacent normal tissues. In addition, increased expression of the lncRNA in CRC patients is associated with increased tumor size and advanced TNM stage. Our subsequent studies demonstrate that HOXA-AS2 knockdown decreased cell proliferation and caused a dramatic decrease in CRC cell colony formation, whereas HOXA-AS2 overexpression has the opposite results. In addition, HOXA-AS2 knockdown promoted significant arrest in the G0/G1-phase and an obvious increase in CRC cell apoptosis. These observations were verified both *in vitro* and *in vivo* by Edu staining, TUNEL staining and immunohistochemical assays, as well as in a mouse xenograft model. These findings suggest that HOXA-AS2 might be a novel clinical molecular marker for the prognosis of CRC patients.

Although lncRNAs exhibit vital biological functions in cancer, the underlying molecular mechanisms involved in lncRNA interactions are complex and diverse.^30, 31, 32^ However, one of the most important mechanisms discovered to date is that lncRNA serves as molecular scaffold, which binds two proteins or RNAs to indirectly exert biological functions. For instance, the lncRNA HOTTIP is associated with the PRC2 and WDR5/MLL1 chromatin-modifying complexes.^33^ The lncRNA HOTAIR binds with PRC2 and the LSD1/CoREST/REST complex.^34^

P21, one of the universal CDK inhibitors, has been reported related to block cell cycle progression at the G0/G1 checkpoint.^35^ Here, we also demonstrate that p21 can act as a tumor suppressor and is silenced by HOXA-AS2 in CRC cells. Meanwhile, HOXA-AS2 can also suppress the expression of KLF2 (a tumor suppressor and the member of KLF family with Cys2/His2 zinc-finger domains).^36^ The p21 and KLF2 mRNA and protein expression was significantly increased after HOXA-AS2 expression was altered in the CRC cells, indicating that p21 and KLF2 may be the possible target of HOXA-AS2. In addition, the nucleocytoplasmic separation experiments demonstrate that HOXA-AS2 RNA is mainly distributed in the nucleus, indicating that HOXA-AS2 may exert transcriptional regulation function.

To further confirm the underlying molecular mechanisms involved, we performed the RIP and RNA-pulldown assays and found that HOXA-AS2 could directly binding with EZH2 and LSD1 in DLD1 and HCT116 cells to silence p21 and KLF2 expression. Furthermore, the results of chromatin immunoprecipitation analysis demonstrated that EZH2 could directly bind to p21 and KLF2 promoter regions and induce H3K27me3 modification, whereas LSD1 could directly couple with their promoter regions and enhance H3K4me2 modifications in the CRC cells. These results revealed that HOXA-AS2 promotes CRC cell proliferation is a manner that is dependent on the regulation of p21 and KLF2 expression by binding to EZH2 and LSD1.

Collectively, we have demonstrated for the first time that HOXA-AS2 expression was upregulated in the CRC tissues and cell lines. HOXA-AS2 may act as an oncogene by significantly promoting proliferation via increasing the G1/S transition and suppressing CRC cell apoptosis *in vitro* and *in vivo*. Furthermore, as the underlying molecular mechanism, HOXA-AS2 may epigenetically silence p21 and KLF2 transcription by binding to EZH2, LSD1. Further insights into the functional and clinical implications of HOXA-AS2 may expedite the appraisal of novel diagnostic or predictive biomarkers for CRC.

## Materials and methods

### Tissue collection and ethics statement

The CRC tissues and normal tissues were collected from 69 patients who underwent surgical resection at the Second Affiliated Hospital of Nanjing Medical University (China) between 2011 and 2014. All patients did not receive any local or systemic treatment before surgery. The pathological stage, grade and nodal status were appraised by an experienced pathologist. All the experiments were approved by the Research Ethics Committee of Nanjing Medical University, China. Written informed consent was obtained from all the patients. The animal experiments were performed with the approval of The Institutional Committee for Animal Research and conformed to national guidelines for the care and use of laboratory animals.

### Cell lines and culture conditions

The human CRC cell lines (HCT116, DLD1, SW480, SW620, HT-29 and LOVO) and the human colonic epithelial cells HCoEpiC were purchased from American Type Culture Collection (Manassas, VA, USA). The cells were cultured in Dulbecco's modified Eagle's medium (DMEM; Invitrogen, Shanghai, China) in humidified air at 37 °C with 5% CO_2_. All media were supplemented with 10% fetal bovine serum (10% FBS), 100 U/ml penicillin and 100 mg/ml streptomycin (Invitrogen).

### RNA extraction and qRT–PCR analyses

RNA extraction and qRT–PCR analyses were performed as described previously.^18^ Each sample was analyzed in triplicate. The primer sequences used for the studies are presented in Additional file 1: Supplementary Table S1.

### Transfection of CRC cells

HOXA-AS2, p21, EZH2, LSD1 and scrambled negative control small interfering RNAs (siRNAs) were purchased from Invitrogen and transfected into cells using Lipofectamine 2000 (Invitrogen, Carlsbad, CA, USA). Plasmid vectors (sh-HOXA-AS2, sh-P21 and empty vector) for transfection were extracted by DNA Midiprep kit (Qiagen, Hilden, Germany) and transfected into cells using Fugene (Roche, Indianapolis, IN, USA). To overexpress HOXA-AS2, the full-length coding sequence for HOXA-AS2 was amplified and subcloned into the pcDNA 3.1(+) vector (Realgene, Shanghai, China) according to the manufacturer's instructions. HCT116, DLD1 and SW480 cells were transfected with a negative control vector or the HOXA-AS2-expressing plasmid according to the manufacturer's protocol. The cells were collected after 48 h for qRT–PCR and western blot analyses. The sequences of the siRNAs are described in Additional file 2: Supplementary Table S2.

### Cell proliferation assays

Forty-eight hours after si-HOXA-AS2 or pCDNA-HOXA-AS2 transfection with a negative control vector, 3000 cells per well were allowed to grow in 96-well plates with five replicate wells. After 6 h of culture as well as at 24, 48, 72 and 96 h after starting the culture, the cells were treated with 100 μg 3-(4,5-dimethylthiazol-2-yl)-2,5-diphenyltetrazolium bromide (MTT) by adding it to the medium. The cells were incubated at 37 °C for an additional 4 h. Then, the medium was removed, and dimethylsulfoxide (DMSO) was added for 10 min to lyse the cells. Finally, the absorbance was measured at 490 nm. All experiments were performed in triplicate.

### Colony-formation and clonogenic assays

The cells were trypsinized into single-cell suspensions 48 h after transfection. For the colony-formation assay, 1000 cells were plated into each well of a six-well plate and maintained in media containing 10% FBS to allow colony formation. The medium was replaced every 4 days. The plates were incubated for 1 to 2 weeks at 37 °C in a 5% CO_2_ atmosphere until colonies formed. The colonies were fixed with methanol and stained with 0.1% crystal violet (Sigma, Darmstadt, Germany) in phosphate-buffered saline for 15 min. The visible colonies were manually counted. All measurements were repeated thrice.

### Flow cytometry

Cell cycle analysis was performed 48 h after transfection with propidium iodide staining as described previously.^18^ Cell apoptosis was analyzed 48 h after transfection by Annexin V and propidium iodide staining as described previously.^18^ All the samples were assayed in triplicate.

### Edu and TUNEL assays

The Edu and TUNEL assays were performed as described previously.^27^ All experiments were performed in triplicate.

### Western blot analysis and antibodies

The cells were lysed using the mammalian protein extraction reagent, RIPA (Beyotime, Shanghai, China), supplemented with a protease inhibitor cocktail (Roche) and PMSF (Roche). Protein were separated by 10% sodium dodecyl sulfate–polyacrylamide gel electrophoresis (SDS–PAGE), transferred to 0.22 mm nitrocellulose membranes (Sigma) and incubated with specific primary antihuman antibodies. The secondary antibody was horseradish peroxidase-conjugated goat anti-rabbit IgG. An ECL chromogenic substrate was used to visualize the bands and the intensity of the bands was quantified by densitometry (Quantity One software; Bio-Rad,Hercules, CA, USA). A GAPDH (#2118, CST, USA) antibody was used as a control. The anti-CDK4 (#12790, CST, USA), p21 (#3698, CST, USA), cleaved caspase-3 (#9664, CST, USA) and cleaved caspase-9 (#9509, CST, USA) (all 1:1000) antibodies were purchased from Cell Signaling Technology, Inc. (CST). The anti-KLF2 (SAB1101046, Sigma) antibody was purchased from Sigma.

### Subcellular fractionation location

The separation of nuclear and cytosolic fractions was performed using the PARIS Kit (Life Technologies, Carlsbad, CA, USA) according to the manufacturer's instructions.

### RIP assay

RIP experiments were performed using the Magna RIP RNA-Binding Protein Immunoprecipitation Kit (Millipore, Billerica, MA, USA) following the manufacturer's instructions. Antibody for RIP assays of EZH2, SUZ12, LSD1 or control IgG were from Millipore.

### RNA pulldown assays

HOXA-AS2 RNAs were *in vitro* transcribed using T7 RNA polymerase (Ambio Life, Cheyenne, WY, USA), which were then purified using the RNeasy Plus Mini Kit (Qiagen) and treated with RNase-free DNase I (Qiagen). Transcribed RNAs were biotin-labeled with the Biotin RNA Labeling Mix (Ambio Life). Positive, negative and Biotinylated RNAs were mixed and incubated with DLD1 cell lysates. Magnetic beads were added to each binding reaction, followed by incubation at room temperature. Then, the beads were washed with washing buffer. The eluted proteins were detected by western blot analysis.

### Chromatin immunoprecipitation assay

The CRC cells were treated with formaldehyde and incubated for 10 mins to generate DNA-protein cross-links. The cell lysates were then sonicated to generate chromatin fragments of 200 to 300 bp and immunoprecipitated with H3K27me3, EZH2, H3K4me2 and LSD1-specific antibody (CST) or IgG as control. Precipitated chromatin DNA was recovered and analyzed by qRT–PCR. The primer sequences used for the studies are presented in Additional file 3: Supplementary Table S3.

### Tumor-formation assay in a nude mouse model

Four-week-old male BALB/c nude mice were obtained from the Shanghai Laboratory Animals Center of the Chinese Academy of Sciences (Shanghai, China). The sample sizes for animal experiments were determined as five mice in sh-HOXA-AS2 groups and three mice in pCDNA-HOXA-AS2 groups. The mice were housed under pathogen-free conditions with a 12 h light/dark schedule, fed an autoclaved diet *ad libitum* and injected subcutaneously with 5 × 10^6^ cells to assess the tumor formation. Neither randomization nor blinding for animal use was performed because we commercially obtained these mice with the same genetic background. Tumor growth was examined every 3 days, and tumor volumes were calculated using the following formula: 0.5 × length × width^2^. Two weeks after cell injection, the mice were killed. The subcutaneous weight of each tumor was measured and the tumors were used for further analysis. The protocol was approved by the Committee on the Ethics of Animal Experiments of the Nanjing Medical University.

### Immunohistochemical analysis

Tumor tissue samples were immunostained for hematoxylin and eosin and Ki-67 as described previously.^17^ The expression was considered to be positive when 50% or more cancer cells were stained.

### Statistical analysis

All the experiments were repeated at least three times with each sample in triplicate. The sample sizes for relevant experiment were determined by power analysis. All data were expressed as the means±s.d. and analyzed using Student's *t*-test to compare two groups of *in vitro* and *in vivo* data using the SPSS 17.0 software program (IBM, Armonk, NY, USA). A value of *P*<0.05 was considered to be statistically significant.

## Figures and Tables

**Table 1:** Correlation between HOXA-AS2 expression and clinicopathological characteristics of CRC patients

*Characteristics*	*Number (*n*=69)*	*Percent*	*HOXA-AS2*		*Chi-squared test* P*-value*
			*High*	*Low*	
Age (years)					0.053
<60	38	55.1	15	23	
⩾60	31	44.9	20	11	
					
Gender					0.797
Male	47	68.1	23	24	
Female	22	31.9	12	10	
					
Maximum tumor size					0.002^a^
<5 cm	37	53.6	12	25	
⩾5 cm	32	46.4	23	9	
					
Location					0.332
Colon	40	58.0	18	22	
Rectum	29	42.0	17	12	
					
Depth of tumor					0.808
T1 and T2	34	49.3	16	17	
T3 and T4	35	50.7	19	16	
					
Tumor stage					0.004^a^
I and II	21	30.4	5	16	
III and IV	48	69.6	30	18	
					
Lymph node metastasis					0.004^a^
Negative	21	30.4	5	16	
Positive	48	69.6	30	18	

a *P*<0.05 was considered significant (Chi-square test between two groups).
