# Supplementary material for: Long noncoding RNA HOXA-AS2 represses P21 and KLF2 expression transcription by binding with EZH2, LSD1 in colorectal cancer
Source: Oncogenesis. 2017 Jan 23;6(1):e288–. doi: 10.1038/oncsis.2016.84 (PMC5294247; doi:10.1038/oncsis.2016.84)
Supplement: Supplementary Information [file oncsis201684x1.pdf]

Long noncoding RNA HOXA-AS2 represses P21 and KLF2 expression transcription by binding with EZH2, LSD1 in colorectal cancer

Supplementary Material

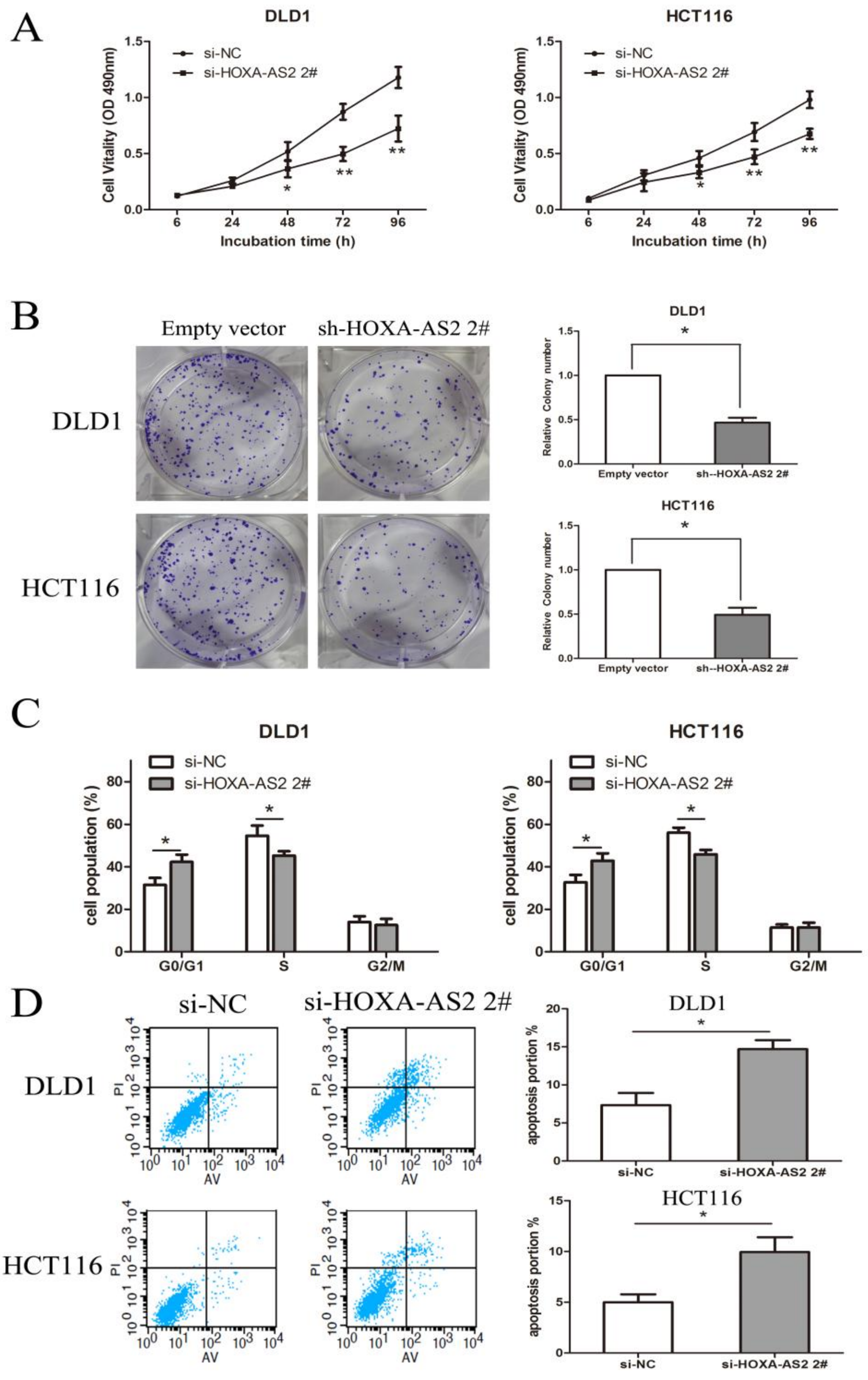

Figure S1. (A) A MTT assay was performed to determine the proliferation of DLD1 and HCT116 cells following treatment with si-HOXA-AS2 2# or si-NC. The data represent the means ± SD from three independent experiments. (B) Colony-forming growth assays were performed to determine the proliferation of CRC cells. The colonies were counted and captured. (C) The bar chart represents the percentage of cells in G0/G1, S, or G2/M phase, as indicated. (D) The percentage of apoptotic cells was determined by flow-cytometric analysis. The data represent the mean ± SD from three independent experiments. *P < 0.05, **P < 0.01.

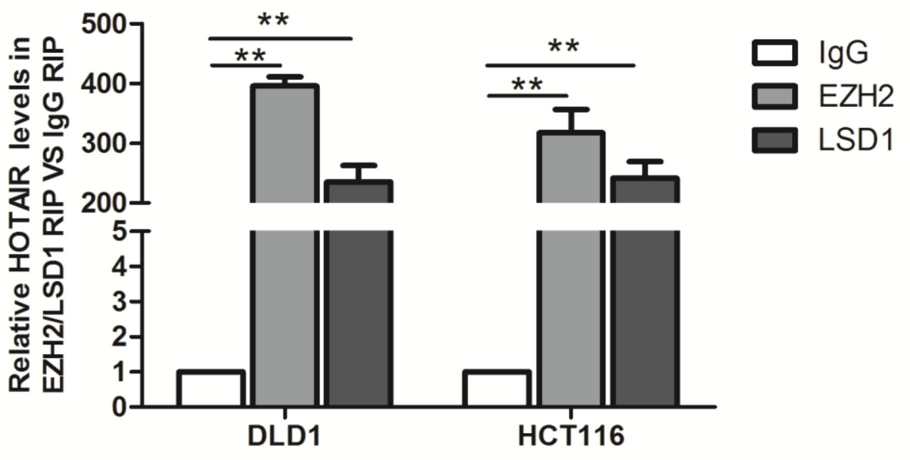

Figure S2. LncRNA HOTAIR was used as positive control to bind with EZH2 and LSD1 in DLD1 and HCT116 cells. The mean values and s.d. were calculated from triplicates of a representative experiment. **P < 0.01.

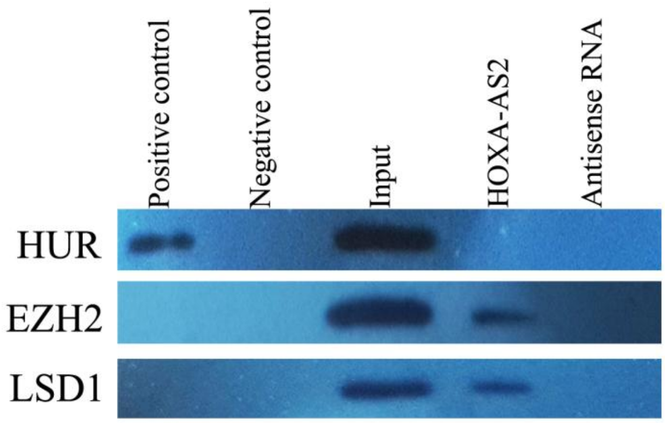

Figure S3. Protein levels in immunoprecipitates were determined by western blot assay. The expression levels of EZH2 and LSD1 protein were presented.
